# 5-Bromo-1*H*-indole-3-carbaldehyde thio­semicarbazone

**DOI:** 10.1107/S160053680801101X

**Published:** 2008-04-26

**Authors:** Mohd. Razali Rizal, Hapipah Mohd. Ali, Seik Weng Ng

**Affiliations:** a Department of Chemistry, University of Malaya, 50603 Kuala Lumpur, Malaysia

## Abstract

In the essentially planar title mol­ecule, C_10_H_9_BrN_4_S, the C=N double bond is in a *trans* configuration. In the crystal structure, the S atom acts as a hydrogen-bond acceptor for the aromatic NH, aliphatic NH and terminal NH_2_ groups of three symmetry-related mol­ecules, forming a weak hydrogen-bonded layer structure.

## Related literature

For a previous synthesis of the title compound, see: Dubey & Babu (2006 ▶). For related literature, see: Doyle *et al.* (1956 ▶); French & Blanz (1966 ▶); Fukukawa *et al.* (1966 ▶); Libermann *et al.* (1953 ▶); Usi (1968 ▶); Weller *et al.* (1954 ▶).
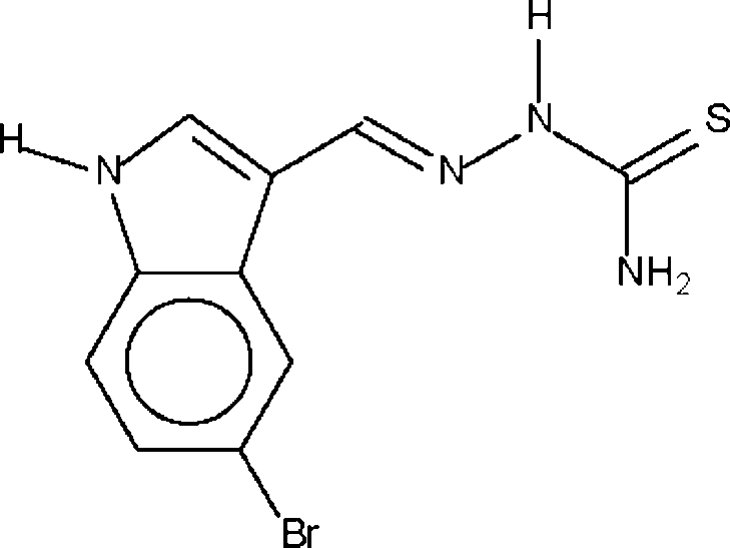


## Experimental

### 

#### Crystal data

C_10_H_9_BrN_4_S
*M* *_r_* = 297.18
Triclinic, 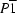

*a* = 6.7731 (2) Å
*b* = 8.7551 (2) Å
*c* = 10.6539 (2) Å
α = 69.280 (1)°
β = 79.969 (1)°
γ = 72.886 (1)°
*V* = 563.00 (2) Å^3^
*Z* = 2
Mo *K*α radiation
μ = 3.81 mm^−1^
*T* = 100 (2) K
0.30 × 0.20 × 0.20 mm

#### Data collection

Bruker SMART APEX diffractometer
Absorption correction: multi-scan (*SADABS*; Sheldrick, 1996 ▶) *T*_min_ = 0.381, *T*_max_ = 0.516 (expected range = 0.344–0.467)
6176 measured reflections
2563 independent reflections
2281 reflections with *I* > 2σ(*I*)
*R*_int_ = 0.025

#### Refinement

*R*[*F* ^2^ > 2σ(*F* ^2^)] = 0.024
*wR*(*F* ^2^) = 0.066
*S* = 1.06
2563 reflections
161 parameters
4 restraints
H atoms treated by a mixture of independent and constrained refinement
Δρ_max_ = 0.36 e Å^−3^
Δρ_min_ = −0.40 e Å^−3^

### 

Data collection: *APEX2* (Bruker, 2007 ▶); cell refinement: *SAINT* (Bruker, 2007 ▶); data reduction: *SAINT*; program(s) used to solve structure: *SHELXS97* (Sheldrick, 2008 ▶); program(s) used to refine structure: *SHELXL97* (Sheldrick, 2008 ▶); molecular graphics: *X-SEED* (Barbour, 2001 ▶); software used to prepare material for publication: *publCIF* (Westrip, 2008 ▶).

## Supplementary Material

Crystal structure: contains datablocks global, I. DOI: 10.1107/S160053680801101X/lh2609sup1.cif

Structure factors: contains datablocks I. DOI: 10.1107/S160053680801101X/lh2609Isup2.hkl

Additional supplementary materials:  crystallographic information; 3D view; checkCIF report

## Figures and Tables

**Table 1:** Hydrogen-bond geometry (Å, °)

*D*—H⋯*A*	*D*—H	H⋯*A*	*D*⋯*A*	*D*—H⋯*A*
N1—H1*n*⋯S1^i^	0.88 (1)	2.60 (2)	3.390 (2)	150 (3)
N3—H3*n*⋯S1^ii^	0.88 (1)	2.65 (1)	3.508 (2)	167 (2)
N4—H4*n*1⋯S1^iii^	0.88 (1)	2.74 (1)	3.569 (2)	158 (2)

Symmetry codes: (i) 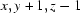; (ii) 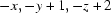; (iii) 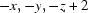.

**Fig. 1.**
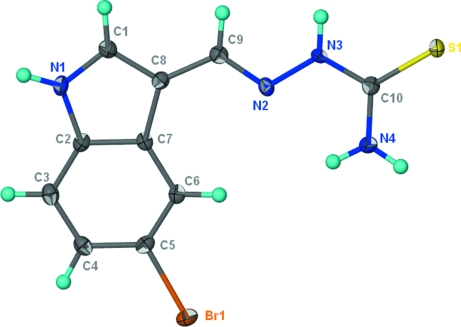
The title molecule drawn using 70% probabilty ellipsoids. Hydrogen atoms are drawn as spheres of arbitrary radius.

## supplementary crystallographic information

### Comment

Indole-3-carboxaldehyde thiosemicarbazone and its substituted analogs possess useful medicinal properties; such activity has been known for a long time (Doyle *et al.*, 1956; French & Blanz, 1966; Fukukawa *et al.*, 1966; Libermann *et al.*, 1953; Usi, 1968; Weller *et al.*, 1954). The compounds, in the form of their metal derivatives, have been assesses for similar activity.

In the title compound (I) (Fig. 1), the double-bonded sulfur atom is a hydrogen-bond acceptor for the aromatic -N-H, aliphatic -N-H and terminal -NH_2_ groups of three adjacent molecules, forming a weak hydrogen-bonded layer structure.

### Experimental

5-Bromoindole-3-carboxaldehyde (0.3 g, 1.3 mmol) and thiosemicarbazide (0.12 g, 1.3 mmol) were heated in ethanol (50 ml) for an hour. The solvent was removed and the product and recrystallized from ethanol.

### Refinement

Carbon-bound H atoms were placed in calculated positions, and were included in the refinement in the riding model approximation. The nitrogen-bound H atoms were located in a difference Fourier map, and were refined with a distance restraint of N–H 0.88±0.01 Å; their temperature factors were freely refined.

### Crystal data

**Table:**

C_10_H_9_BrN_4_S	*Z* = 2
*M**_r_* = 297.18	*F*_000_ = 296
Triclinic, *P*1	*D*_x_ = 1.753 Mg m^−^^3^
Hall symbol: -P 1	Mo *K*α radiation λ = 0.71073 Å
*a* = 6.7731 (2) Å	Cell parameters from 6604 reflections
*b* = 8.7551 (2) Å	θ = 4.0–28.3º
*c* = 10.6539 (2) Å	µ = 3.81 mm^−^^1^
α = 69.280 (1)º	*T* = 100 (2) K
β = 79.969 (1)º	Block, yellow
γ = 72.886 (1)º	0.30 × 0.20 × 0.20 mm
*V* = 563.00 (2) Å^3^	

### Data collection

**Table:**

Bruker SMART APEX diffractometer	2563 independent reflections
Radiation source: fine-focus sealed tube	2281 reflections with *I* > 2σ(*I*)
Monochromator: graphite	*R*_int_ = 0.025
*T* = 100(2) K	θ_max_ = 27.5º
ω scans	θ_min_ = 2.1º
Absorption correction: multi-scan(SADABS; Sheldrick, 1996)	*h* = −6→8
*T*_min_ = 0.381, *T*_max_ = 0.516	*k* = −11→11
6176 measured reflections	*l* = −13→13

### Refinement

**Table:**

Refinement on *F*^2^	Secondary atom site location: difference Fourier map
Least-squares matrix: full	Hydrogen site location: inferred from neighbouring sites
*R*[*F*^2^ > 2σ(*F*^2^)] = 0.024	H atoms treated by a mixture of independent and constrained refinement
*wR*(*F*^2^) = 0.066	*w* = 1/[σ^2^(*F*_o_^2^) + (0.0383*P*)^2^ + 0.1*P*] where *P* = (*F*_o_^2^ + 2*F*_c_^2^)/3
*S* = 1.06	(Δ/σ)_max_ = 0.001
2563 reflections	Δρ_max_ = 0.36 e Å^−^^3^
161 parameters	Δρ_min_ = −0.40 e Å^−^^3^
4 restraints	Extinction correction: none
Primary atom site location: structure-invariant direct methods	

### Fractional atomic coordinates and isotropic or equivalent isotropic displacement parameters (Å^2^)

**Table:**

	*x*	*y*	*z*	*U*_iso_*/*U*_eq_	
Br1	0.51693 (4)	0.13022 (2)	0.31237 (2)	0.02102 (9)	
S1	0.01187 (9)	0.22591 (6)	1.06142 (5)	0.01534 (12)	
N1	0.2717 (3)	0.8577 (2)	0.27383 (17)	0.0153 (4)	
N2	0.1383 (3)	0.4698 (2)	0.68281 (16)	0.0116 (3)	
N3	0.0718 (3)	0.4342 (2)	0.81750 (16)	0.0118 (3)	
N4	0.1492 (3)	0.1567 (2)	0.83268 (18)	0.0169 (4)	
C1	0.1846 (3)	0.8475 (3)	0.4006 (2)	0.0151 (4)	
H1	0.1266	0.9415	0.4320	0.018*	
C2	0.3383 (3)	0.6981 (2)	0.2626 (2)	0.0122 (4)	
C3	0.4355 (3)	0.6471 (3)	0.1529 (2)	0.0142 (4)	
H3	0.4641	0.7265	0.0687	0.017*	
C4	0.4889 (3)	0.4765 (3)	0.1711 (2)	0.0131 (4)	
H4	0.5573	0.4359	0.0990	0.016*	
C5	0.4422 (3)	0.3633 (2)	0.2960 (2)	0.0127 (4)	
C6	0.3464 (3)	0.4108 (2)	0.40581 (19)	0.0118 (4)	
H6	0.3184	0.3300	0.4893	0.014*	
C7	0.2917 (3)	0.5832 (2)	0.38933 (19)	0.0109 (4)	
C8	0.1921 (3)	0.6825 (2)	0.4770 (2)	0.0120 (4)	
C9	0.1249 (3)	0.6266 (2)	0.6176 (2)	0.0127 (4)	
H9	0.0693	0.7074	0.6633	0.015*	
C10	0.0822 (3)	0.2737 (2)	0.8937 (2)	0.0123 (4)	
H1N	0.254 (5)	0.953 (2)	0.207 (2)	0.028 (7)*	
H3N	0.037 (4)	0.510 (2)	0.858 (2)	0.013 (6)*	
H4N1	0.137 (4)	0.0533 (17)	0.876 (2)	0.023 (7)*	
H4N2	0.173 (5)	0.190 (4)	0.7452 (11)	0.035 (8)*	

### Atomic displacement parameters (Å^2^)

**Table:**

	*U*^11^	*U*^22^	*U*^33^	*U*^12^	*U*^13^	*U*^23^
Br1	0.02971 (15)	0.01243 (12)	0.02039 (12)	−0.00512 (9)	0.00410 (9)	−0.00765 (8)
S1	0.0217 (3)	0.0111 (2)	0.0101 (2)	−0.0029 (2)	0.00152 (19)	−0.00200 (18)
N1	0.0190 (10)	0.0104 (8)	0.0119 (8)	−0.0022 (7)	0.0015 (7)	−0.0005 (6)
N2	0.0105 (8)	0.0144 (8)	0.0100 (8)	−0.0056 (6)	0.0021 (6)	−0.0034 (6)
N3	0.0154 (9)	0.0112 (8)	0.0091 (7)	−0.0032 (7)	0.0020 (6)	−0.0051 (6)
N4	0.0239 (10)	0.0117 (8)	0.0144 (8)	−0.0050 (7)	0.0044 (7)	−0.0056 (7)
C1	0.0176 (11)	0.0133 (9)	0.0127 (9)	−0.0021 (8)	0.0005 (8)	−0.0045 (8)
C2	0.0120 (10)	0.0108 (9)	0.0134 (9)	−0.0032 (7)	−0.0023 (8)	−0.0025 (7)
C3	0.0121 (10)	0.0167 (10)	0.0119 (9)	−0.0036 (8)	−0.0015 (8)	−0.0022 (7)
C4	0.0109 (10)	0.0174 (10)	0.0111 (9)	−0.0028 (8)	−0.0020 (7)	−0.0049 (7)
C5	0.0113 (10)	0.0106 (9)	0.0165 (9)	−0.0031 (7)	−0.0014 (8)	−0.0042 (7)
C6	0.0102 (10)	0.0124 (9)	0.0117 (9)	−0.0030 (7)	−0.0017 (7)	−0.0021 (7)
C7	0.0090 (10)	0.0132 (9)	0.0103 (9)	−0.0028 (7)	−0.0012 (7)	−0.0032 (7)
C8	0.0114 (10)	0.0116 (9)	0.0131 (9)	−0.0035 (8)	−0.0011 (7)	−0.0037 (7)
C9	0.0107 (10)	0.0137 (9)	0.0133 (9)	−0.0024 (8)	−0.0003 (8)	−0.0049 (7)
C10	0.0103 (10)	0.0129 (9)	0.0130 (9)	−0.0029 (7)	0.0001 (7)	−0.0038 (7)

### Geometric parameters (Å, °)

**Table:**

Br1—C5	1.9032 (19)	C1—H1	0.9500
S1—C10	1.699 (2)	C2—C3	1.388 (3)
N1—C1	1.359 (3)	C2—C7	1.418 (3)
N1—C2	1.378 (3)	C3—C4	1.379 (3)
N1—H1N	0.878 (10)	C3—H3	0.9500
N2—C9	1.284 (3)	C4—C5	1.399 (3)
N2—N3	1.378 (2)	C4—H4	0.9500
N3—C10	1.339 (3)	C5—C6	1.372 (3)
N3—H3N	0.876 (10)	C6—C7	1.398 (3)
N4—C10	1.331 (3)	C6—H6	0.9500
N4—H4N1	0.880 (10)	C7—C8	1.447 (3)
N4—H4N2	0.873 (10)	C8—C9	1.437 (3)
C1—C8	1.376 (3)	C9—H9	0.9500
			
C1—N1—C2	109.25 (17)	C3—C4—H4	120.1
C1—N1—H1N	122.5 (19)	C5—C4—H4	120.1
C2—N1—H1N	126.2 (18)	C6—C5—C4	123.94 (18)
C9—N2—N3	115.12 (17)	C6—C5—Br1	118.76 (15)
C10—N3—N2	119.25 (16)	C4—C5—Br1	117.30 (15)
C10—N3—H3N	117.5 (16)	C5—C6—C7	117.04 (18)
N2—N3—H3N	122.8 (16)	C5—C6—H6	121.5
C10—N4—H4N1	119.9 (17)	C7—C6—H6	121.5
C10—N4—H4N2	118.1 (19)	C6—C7—C2	119.09 (17)
H4N1—N4—H4N2	120 (3)	C6—C7—C8	134.17 (18)
N1—C1—C8	110.69 (18)	C2—C7—C8	106.75 (17)
N1—C1—H1	124.7	C1—C8—C9	124.92 (18)
C8—C1—H1	124.7	C1—C8—C7	105.86 (17)
N1—C2—C3	129.70 (18)	C9—C8—C7	129.10 (18)
N1—C2—C7	107.45 (17)	N2—C9—C8	121.41 (18)
C3—C2—C7	122.85 (18)	N2—C9—H9	119.3
C4—C3—C2	117.27 (18)	C8—C9—H9	119.3
C4—C3—H3	121.4	N4—C10—N3	117.36 (18)
C2—C3—H3	121.4	N4—C10—S1	122.58 (16)
C3—C4—C5	119.81 (18)	N3—C10—S1	120.06 (15)
			
C9—N2—N3—C10	−179.30 (19)	C3—C2—C7—C6	0.3 (3)
C2—N1—C1—C8	0.5 (3)	N1—C2—C7—C8	0.4 (2)
C1—N1—C2—C3	180.0 (2)	C3—C2—C7—C8	179.9 (2)
C1—N1—C2—C7	−0.6 (2)	N1—C1—C8—C9	176.0 (2)
N1—C2—C3—C4	178.9 (2)	N1—C1—C8—C7	−0.2 (3)
C7—C2—C3—C4	−0.6 (3)	C6—C7—C8—C1	179.4 (2)
C2—C3—C4—C5	0.8 (3)	C2—C7—C8—C1	−0.2 (2)
C3—C4—C5—C6	−0.8 (3)	C6—C7—C8—C9	3.4 (4)
C3—C4—C5—Br1	179.01 (16)	C2—C7—C8—C9	−176.2 (2)
C4—C5—C6—C7	0.6 (3)	N3—N2—C9—C8	178.80 (19)
Br1—C5—C6—C7	−179.25 (15)	C1—C8—C9—N2	−179.4 (2)
C5—C6—C7—C2	−0.3 (3)	C7—C8—C9—N2	−4.0 (4)
C5—C6—C7—C8	−179.8 (2)	N2—N3—C10—N4	−2.8 (3)
N1—C2—C7—C6	−179.18 (18)	N2—N3—C10—S1	177.10 (15)

### Hydrogen-bond geometry (Å, °)

**Table:**

*D*—H···*A*	*D*—H	H···*A*	*D*···*A*	*D*—H···*A*
N1—H1*n*···S1^i^	0.88 (1)	2.60 (2)	3.390 (2)	150 (3)
N3—H3*n*···S1^ii^	0.88 (1)	2.65 (1)	3.508 (2)	167 (2)
N4—H4*n*1···S1^iii^	0.88 (1)	2.74 (1)	3.569 (2)	158 (2)

Symmetry codes: (i) *x*, *y*+1, *z*−1; (ii) −*x*, −*y*+1, −*z*+2; (iii) −*x*, −*y*, −*z*+2.

## References

[bb1] Barbour, L. J. (2001). *J. Supramol. Chem.* **1**, 189–191.

[bb2] Bruker (2007). *APEX2* and *SAINT* Bruker AXS Inc., Madison, Wisconsin, USA.

[bb3] Doyle, F. P., Ferrier, W., Holland, D. O., Mehta, M. D. & Nayler, J. H. C. (1956). *J. Chem. Soc.* pp. 2853–2857.

[bb4] Dubey, P. K. & Babu, B. (2006). *Ind. J. Heterocycl. Chem.* **15**, 209–219.

[bb5] French, F. A. & Blanz, E. J. (1966). *J. Med. Chem.* **9**, 585–589. 10.1021/jm00322a032 5968028

[bb6] Fukukawa, F., Isao, Y., Seno, T., Sasaki, M., Naito, M. & Shunji, T. (1966). *Yakaguka Zasshi*, **86**, 801–804.

[bb7] Libermann, D., Moyeux, M., Rouaix, A., Maillard, J., Hengl, L., Himbert, J. & Theraplix, M. (1953). *Bull. Soc. Chim. Fr.* pp. 957–962.

[bb8] Sheldrick, G. M. (1996). *SADABS.* University of Göttingen, Germany.

[bb9] Sheldrick, G. M. (2008). *Acta Cryst.* A**64**, 112–122. 10.1107/S0108767307043930 18156677

[bb10] Usi, Y. (1968). *Ann. Rep. Takeda Res. Lab.* **27**, 144–158.

[bb11] Weller, L. E., Sell, H. M. & Gotshall, R. Y. (1954). *J. Am. Chem. Soc.* **76**, 1959.

[bb12] Westrip, S. P. (2008). *publCIF* In preparation.

